# Targeting PP2A-dependent autophagy enhances sensitivity to ruxolitinib in JAK2^V617F^ myeloproliferative neoplasms

**DOI:** 10.1038/s41408-023-00875-x

**Published:** 2023-07-10

**Authors:** Charly Courdy, Loïc Platteeuw, Charlotte Ducau, Isabelle De Araujo, Emeline Boet, Ambrine Sahal, Estelle Saland, Valérie Edmond, Suzanne Tavitian, Sarah Bertoli, Pierre Cougoul, Fanny Granat, Laura Poillet, Caroline Marty, Isabelle Plo, Jean-Emmanuel Sarry, Stéphane Manenti, Véronique Mansat-De Mas, Carine Joffre

**Affiliations:** 1 Centre de Recherches en Cancérologie de Toulouse (CRCT), INSERM UMR1037, CNRS UMR 5071, Université de Toulouse, Toulouse, France; 2 Equipe labellisée La Ligue contre le Cancer 2018, Toulouse, France; 3 INSERM UMR1287, Gustave Roussy, Université Paris-Saclay, Villejuif, France; 4 Service d’hématologie, Centre Hospitalier Universitaire de Toulouse, Institut Universitaire du Cancer de Toulouse-Oncopole, Université de Toulouse III Paul Sabatier, Toulouse, France; 5 Service de médecine interne, Centre Hospitalier Universitaire de Toulouse, Institut Universitaire du Cancer de Toulouse-Oncopole, Toulouse, France; 6 Laboratoire d’Hématologie, Centre Hospitalier Universitaire de Toulouse, Institut Universitaire du Cancer de Toulouse-Oncopole, Université de Toulouse III Paul Sabatier, Toulouse, France

**Keywords:** Myeloproliferative disease, Cell signalling

## Abstract

The Janus kinase 2 (JAK2)-driven myeloproliferative neoplasms (MPNs) are chronic malignancies associated with high-risk complications and suboptimal responses to JAK inhibitors such as ruxolitinib. A better understanding of cellular changes induced by ruxolitinib is required to develop new combinatory therapies to improve treatment efficacy. Here, we demonstrate that ruxolitinib induced autophagy in JAK2^V617F^ cell lines and primary MPN patient cells through the activation of protein phosphatase 2A (PP2A). Inhibition of autophagy or PP2A activity along with ruxolitinib treatment reduced proliferation and increased the death of JAK2^V617F^ cells. Accordingly, proliferation and clonogenic potential of JAK2^V617F^-driven primary MPN patient cells, but not of normal hematopoietic cells, were markedly impaired by ruxolitinib treatment with autophagy or PP2A inhibitor. Finally, preventing ruxolitinib-induced autophagy with a novel potent autophagy inhibitor Lys05 improved leukemia burden reduction and significantly prolonged the mice’s overall survival compared with ruxolitinib alone. This study demonstrates that PP2A-dependent autophagy mediated by JAK2 activity inhibition contributes to resistance to ruxolitinib. Altogether, our data support that targeting autophagy or its identified regulator PP2A could enhance sensitivity to ruxolitinib of JAK2^V617F^ MPN cells and improve MPN patient care.

## Introduction

The Janus kinase 2 (JAK2) V617F mutation is an activating JAK2 tyrosine kinase mutation detected in the majority of MPN patients with polycythemia vera (PV), and in half of those with essential thrombocytemia (ET) and primary myelofibrosis (MF) [1–4]. Its discovery in 2005 has been decisive not only for diagnosis, but also for the understanding of myeloproliferative neoplasms (MPN) pathogenesis, and has provided a rationale for the development of targeted therapy approaches.

Current JAK2 inhibitors approved for the treatment of MPN patients are ruxolitinib and, more recently, fedratinib. These two type I inhibitors target the active conformation of the JAK2 ATP binding site, thereby interfering with JAK2 catalytic activity [5]. Ruxolitinib, a JAK1/JAK2 inhibitor, represents a clinical standard of care for patients with intermediate or high-risk MF [6], and has also been approved for patients with PV that are resistant or intolerant to hydroxyurea [7]. Ruxolitinib treatment shows important benefits for MPN patients, characterized by a reduction of splenomegaly, symptom burden, blood counts and inflammation in MF and PV and even increased survival in MF [8, 9]. However, since this inhibitor targets both wild type and mutated JAK, it has important toxic side effects, as well as a limited efficacy. Indeed, decrease in the clone size, measured by a reduction in mutant allele burden, is usually modest and the response can be lost during prolonged exposure [6]. Several mechanisms of resistance to JAK2 inhibitors have already been identified, such as JAK-STAT reactivation by JAK family heterodimer formation [10] or additional mechanisms reviewed in Meyer et al. [11], but pre-clinical application for different therapeutic options is still undergoing. Therefore, a better understanding of the molecular mechanisms involved is required to overcome this resistance and to develop new and more effective therapeutic approaches.

Autophagy is a catabolic process that degrades and recycles intracellular components, such as damaged organelles and macromolecules, as an adaptative survival mechanism activated during cellular stresses. However, autophagy is not only a lysosomal degradation pathway; it is also a regulator of cellular metabolism as well as a mechanism involved in tumor initiation/progression and therapeutic resistance in many cancers [12]. Indeed, various oncogenes, such as BCR-ABL1 or FLT3-ITD, were reported to support high levels of basal autophagy sustaining cell proliferation and leukemogenesis in chronic myeloid leukemia (CML) or in acute myeloid leukemia (AML), respectively [13–15]. Moreover, in CML expressing BCR-ABL1, autophagy is induced following in vitro tyrosine kinase inhibitor (TKI) treatment and its pharmacological inhibition potentiates treatment efficacy [16]. These results suggest that autophagy may be involved not only in leukemogenesis but also in the resistance of cells to TKI inhibitors, and represents a potentially attractive approach in treating these hematological malignancies especially in MPN. Indeed, it was recently shown that ruxolitinib induces autophagy in a JAK2^V617F^-mutated cell line and that the pharmacological inhibition of autophagy increased ruxolitinib-induced apoptosis in this model [17, 18]. However, the contribution of autophagy to ruxolitinib resistance in in vivo models and in MPN patient cells, as well as the underlying mechanisms, are currently unknown.

Here, we show that ruxolitinib induces a cytoprotective autophagy involving the phosphatase PP2A signaling pathway. Moreover, autophagy or PP2A inhibition enhances sensitivity to ruxolitinib treatment. Thus, this study reports autophagy, or, more precisely, PP2A-dependent autophagy, as a new therapeutic target able to sensitize cells to ruxolitinib treatment and to improve patient care.

## Materials and methods

### Cell lines culture and treatments

The human cell lines HEL (expressing JAK2^V617F^), SET-2 (expressing both JAK2^V617F^ and JAK2^WT^) and HL-60 (expressing JAK2^WT^) were purchased from the Leibniz Institute DSMZ-German Collection of Microorganisms and Cell Cultures (Braunschweig, Germany). Cells were grown in RPMI 1640 medium with Glutamax (Gibco, Life Technologies, Carlsbad, CA, USA) supplemented with 10% fetal bovine serum (Gibco).

Ruxolitinib and LB-100 were purchased from Selleck Chemicals (Houston, TX, USA). These drugs were used at a concentration of 1 µM and 2.5 µM, respectively, apart from in clonogenic assays where they were used at 250 nM and 1 µM. Bafilomycin (25 nM) was purchased from Invivogen (Toulouse, France). Chloroquine and Lys05 were purchased from Sigma-Aldrich (Saint-Louis, MO, USA). Chloroquine was used at 20 µM in cell lines, 10 µM in primary cultures and at 1 µM for clonogenic assays. Lys05 was used in vitro at a concentration of 5 µM, except for in clonogenic assays, in which it was used at 1 µM. SAR405 (3 µM) and Okadaic acid (OA, 5 nM) were purchased from APExBIO (Houston, TX, USA) and Cell Signaling Technology (Danvers, MA, USA), respectively.

### Patient samples

Primary MPN patient cells have been obtained after informed consent from HIMIP collection (BB-0033-00060). In accordance with French law, HIMIP collection has been declared to the Ministry of Higher Education and Research (DC 2008-307 collection 1) and obtained a transfer agreement (AC 2008-129) after approbation by the “Comité de Protection des Personnes Sud-Ouest et Outremer II” (ethical committee). Clinical and biological annotations of the samples have been declared to the CNIL (Comité National Informatique et Libertés, i.e., National Committee for Data Processing and Liberties).

Normal PBMC were obtained from blood samples of healthy donors (EFS, Etablissement Français du Sang, Toulouse, France). EFS is a governmental agency collecting and delivering blood products. All procedures in use by the EFS are defined by the Law. Briefly, mononuclear cells were separated by Ficoll-Paque density gradient centrifugation and incubated in Erythrocytes Lysis buffer (Qiagen, Hilden, Germany) to remove the remaining red blood cells. CD34+ cells were then sorted using EasySep^TM^ Human CD34 positive selection kit II (17856, Stemcell Technologies, Vancouver, Canada) according to the manufacturer’s instructions. Before immunofluorescence analysis, cells were cultured for 24 h in Iscove’s Modified Dulbecco’s Medium (IMDM, Gibco) supplemented with 20% fetal bovine serum.

### Antibodies and reagents

The following antibodies from Cell Signaling Technology were used: rabbit antibodies against LC3B (2775), P-STAT5 Y694 (9351), STAT5 (9363), P-P70S6K T421/S424 (9204), P70S6K (2708), P-ULK1 S757 (14202), P-ULK1 S638 (14205), P-ULK1 S555 (5869), ULK1 (6439), P-ATG14 S29 (92340) and ATG14 (5504). Mouse antibodies against P-AMPK T172/T183 (sc-101630) and AMPK (sc-25792) were purchased from Santa Cruz Biotechnology (Dallas, TX, USA). Mouse anti-actin (MAB1501) was purchased from Millipore (Burlington, MA, USA). Rabbit anti-LC3B (PM036) from MBL (Woburn, MA, USA) was used for immunofluorescence analysis. Anti-rabbit and anti-mouse secondary antibodies labeled with horseradish peroxidase were purchased from Promega (Madison, WI, USA) and anti-rabbit-AlexaFluor488 was purchased from Invitrogen (Carlsbad, CA, USA). For flow cytometry analysis, the antibody anti-hCD45-V450, Annexin-V-FITC, and Cell Viability Stain from BD Biosciences (Franklin Lakes, NJ, USA) were used.

### Western blot analysis

Proteins were separated using 4–12% gradient poly-acrylamide SDS-PAGE gels (Life Technologies) and electrotransferred to 0.2 µm nitrocellulose membranes (GE Healthcare, Chicago, IL, USA). After blocking with Tris-Buffered Saline with 0.1% Tween and 3% bovine serum albumin, membranes were incubated overnight at 4 °C under continuous shaking with the appropriate primary antibodies. Primary antibodies were detected using the appropriate secondary antibodies coupled with horseradish peroxidase. Immunoreactive bands were visualized by enhanced chemiluminescence with a Syngene camera. Densitometric analyses of immunoblots were performed using the GeneTools software.

### Immunofluorescence analysis

Cells were seeded onto glass slides coated with cell-tak (Corning, Corning, NY, USA), then fixed in 4% paraformaldehyde for 10 min. After PBS washes, cells were incubated in 0.01% saponin with 3% bovine serum albumin for 30 min and then incubated with anti-LC3B antibody (1/800) for 40 min. Cells were then washed before incubation with secondary antibody (1/800) for 30 min, followed by washes using PBS and distilled H_2_O and mounting in ProLong^TM^ Gold antifade medium with DAPI (4′6-diamidino-2-phenylindole, Invitrogen). Images were acquired using a confocal Zeiss LSM 780. For quantifications, fields were arbitrarily chosen based on DAPI staining, and the number of LC3B dots per cell was determined with Image J software.

### Flow cytometry analysis

To determine the number of apoptotic and dead cells, cells were washed and resuspended in 100 µl of Annexin-V binding buffer plus 2 µl of Annexin-V-FITC and 5 µl of Cell Viability Stain. Data were acquired on a Macsquant flow cytometer (Miltenyi Biotec, Bergisch Gladbach, Germany) and analyzed with FlowJo v10 software (Tree Star Inc., Ashland, OR, USA).

### Clonogenic assays

Sorted human peripheral blood CD34+ cells were plated in duplicate at 3000 cells per ml of methylcellulose medium (H4230, Stemcell Technologies) supplemented with 3 U/ml erythropoietin, 50 ng/ml Stem Cell Factor and 25 ng/ml interleukin-3. Erythroid colonies were scored on day 14.

### Tumor xenografts into NSG mice

Animals were used for transplantation of HEL cell lines in accordance with a protocol reviewed and approved by the Institutional Animal Care and Use Committee of Région Midi-Pyrénées (France). *NOD/LtSz-SCID/IL-2Rγchain null* (NSG) mice were produced at the Genotoul Anexplo platform in Toulouse (France) using breeders obtained from Charles River Laboratories (Wilmington, NC, USA). Mice were housed in sterile conditions using HEPA-filtered micro-isolators, and fed with irradiated food and sterile water. Eight-week-old mice were sublethally treated with busulfan (20 mg/kg) 24 h before the injection of HEL cells. Cells were washed in PBS, and suspended in Hanks’ Balanced Salt Solution (HBSS) at a final concentration of 2 × 10^6^ cells per 200 μL of HBSS per mouse for tail vein injection. Mice were then treated each day from day 3 post-cell injection by oral gavage with a vehicle or with 120 mg/kg of ruxolitinib and by intraperitoneal injections with a vehicle or Lys05 at 32 mg/kg. Ruxolitinib was solubilized in DMSO and extemporaneously diluted in water containing 0.5% methylcellulose and 0.1% Tween80 before administration to mice. Lys05 was solubilized in PBS. Mice were sacrificed after 14 days to harvest human cells from murine bone marrow. Engraftment in bone marrow was analyzed by flow cytometry using hCD45-V450 marker. Mice survival time was also determined. Survival significance was determined by Kaplan–Meier curve and log-rank test. No statistical methods were required to determine the sample size. Randomization and blinding were not necessary for this study.

### PP2A phosphatase activity assay

PP2A phosphatase activity was determined using the PP2Ac immunoprecipitation phosphatase assay kit (17-313, Millipore). Briefly, cells were lysed with a buffer containing 2% CHAPS, 20 mM Tris-HCl pH7.4, 137 mM NaCl, 2 mM EDTA, 10% Glycerol and protease inhibitors (Roche, Basel, Switzerland). A total of 100 µg of protein lysate, 4 µg of PP2Ac antibody and 25 µl of protein A-agarose beads were incubated for PP2Ac immunoprecipitation at 4 °C for 2 h. Immunoprecipitates were then used in the phosphatase reaction according to the manufacturer’s instructions. After colorimetric assay, results were normalized to the PP2Ac immunoprecipitation efficacy assessed by western blot.

### Statistical analysis

Data from at least three independent experiments are reported as means ± standard error of the mean. Unpaired two-tailed Student’s t-tests with Welch’s correction were carried out with Prism 8 software (GraphPad Software Inc., San Diego, CA, USA).

## Results

### Ruxolitinib enhances autophagy in JAK2^V617F^ cells

Although recent data from the literature indicate that ruxolitinib treatment induces autophagy in cell lines harboring the JAK2^V617F^ mutation in vitro [17, 18], compelling data about the impact of ruxolitinib on autophagy in MPN patients’ cells and in vivo models are still needed. Moreover, the molecular mechanisms underlying the induction of ruxolitinib-dependent autophagy remain unknown.

**Fig. 1:**
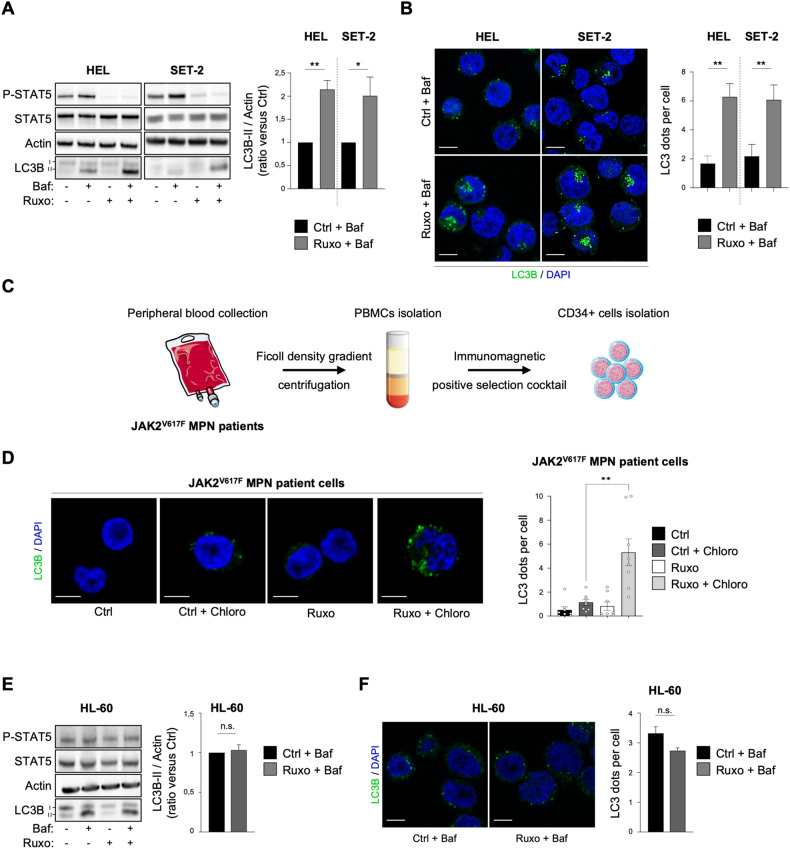
Ruxolitinib increases autophagy in JAK2^V617F^ cells. **A**, **B** HEL and SET-2 cells were treated with ruxolitinib (1 µM) for 2 hours in the presence or absence of bafilomycin (25 nM) to monitor the autophagy flux. **A** Cells were processed for western blot analysis of P-STAT5, STAT5 and LC3B. Actin was used as a loading control. Graphs represent the LC3B-II/actin ratio obtained by densitometric analysis (*n* = 4 ± sem). **B** Cells were stained for LC3B and analyzed by confocal microscopy. Graphs represent the number of LC3B dots per cell (*n* = 4 ± sem). Scale bar: 10 µm. **C** Mononuclear cells from peripheral blood samples from JAK2^V617F^-positive MPN patients were separated by Ficoll-Hypaque density gradient centrifugation. CD34+ progenitors were then sorted using an immunomagnetic positive selection kit and processed for further experiments. **D** Patients’ cells obtained in **C** were treated or not with chloroquine (20 µM) over 16 hours to monitor autophagy flux in combination with ruxolitinib (1 µM) for 2 h and stained for LC3B. Representative confocal pictures are shown and histograms represent the number of LC3B dots per cell (*n* = 8 ± sem). Scale bar: 10 µm. **E**, **F** JAK2^WT^ HL-60 cells were treated with ruxolitinib (1 µM) for 2 hours in the presence or absence of bafilomycin (25 nM) to monitor autophagy flux. **E** Cells were processed for western blot analysis of P-STAT5, STAT5 and LC3B. Actin was used as a loading control. Graphs represent the LC3B-II/actin ratio obtained by densitometric analysis (*n* = 3 ± sem). **F** Cells were stained for LC3B and analyzed by confocal microscopy. Graphs represent the number of LC3B dots per cell (*n* = 3). Scale bar: 10 µm.

Therefore, in order to investigate the effect of ruxolitinib on autophagy in JAK2^V617F^ models, we first treated human JAK2^V617F^ cells from two HEL and SET-2 cell lines and eight primary PV patients with ruxolitinib (1 µM) for 2 h. Bafilomycin (baf) and chloroquine, both autophagy inhibitors, were used to monitor the autophagic flux. As expected and as shown in Fig. 1A, ruxolitinib reduced STAT5 phosphorylation level in JAK2 cell lines. In addition, in association with bafilomycin, ruxolitinib increased lipidated LC3 (LC3B-II) accumulation, an autophagosomal membrane marker, compared to bafilomycin alone in HEL and SET-2 cell lines (Fig. 1A). This effect lasted even following 24 hours of ruxolitinib treatment (Supplementary Fig. 1A). Accordingly, the average number of autophagosomes per cell was significantly increased upon ruxolitinib treatment compared to the control in both cell lines as measured by LC3B immunofluorescence (Fig. 1B). We confirmed these results on JAK2^V617F^ samples from MPN patients (Supplementary Table 1). We isolated cells from phlebotomy followed by CD34+ purification as described in Fig. 1C and we treated them with ruxolitinib with or without chloroquine. Ruxolitinib combined with chloroquine led to an increase in autophagosome number in JAK2^V617F^ primary cells compared to treatments alone (Fig. 1D), as previously observed in JAK2^V617F^ cell lines. Interestingly, in JAK2 wild type (WT) HL-60 AML cell line, ruxolitinib modified neither P-STAT5 level, nor conversion of LC3B-I into LC3B-II (Fig. 1E) or the number of autophagosomes (Fig. 1F), indicating that for ruxolitinib to have an effect on autophagy requires JAK2 inhibition. In order to definitively rule out ruxolitinib off-target effects, we investigated the impact of two other JAK2 inhibitors on autophagy response [19]: fedratinib, a clinically-used type I inhibitor like ruxolitinib, and CHZ868, a type II inhibitor, which target the ATP binding site under active and inactive conformation, respectively. Similar to ruxolitinib, fedratinib and CHZ868 both induced LC3B-II accumulation when combined with bafilomycin, compared to bafilomycin alone in JAK2^V617F^ cells (Supplementary Figure 1B).

Altogether, these complementary biochemical and cellular data demonstrate that autophagy is induced early upon ruxolitinib treatment, and more generally upon JAK2 inhibition in JAK2^V617F^ models (i.e., cell lines and primary cells).

### Targeting autophagy increases ruxolitinib efficacy in JAK2^V617F^ cells

**Fig. 2:**
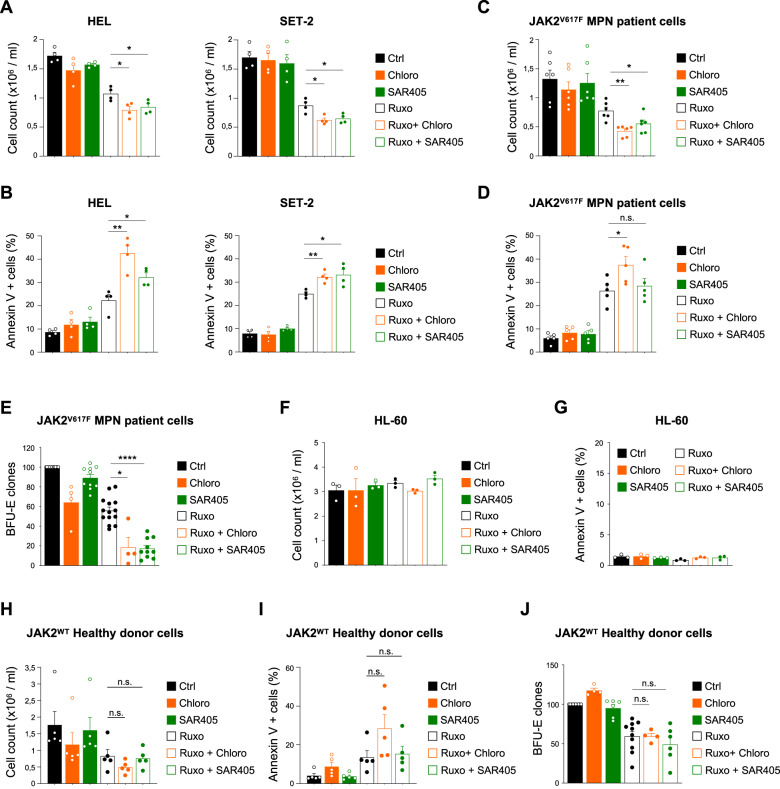
Autophagy inhibition enhances ruxolitinib efficacy in JAK2^V617F^ cells in vitro. **A**, **B** HEL and SET-2 cells were treated or not with ruxolitinib (1 µM) in the presence or absence of chloroquine (20 µM) or SAR405 (3 µM). After 3 days, the number of cells was assessed by trypan blue exclusion counting (*n* = 4 ± sem). **A** and the percentage of cell death was determined by Annexin-V labeling and flow cytometry analysis (*n* = 4 ± sem) **B**. **C**, **D** CD34+ cells obtained from JAK2^V617F^-positive MPN patient samples were treated or not with ruxolitinib (1 µM) in the presence or absence of chloroquine (10 µM) or SAR405 (3 µM). After 2 days, the number of cells was assessed by trypan blue exclusion counting (*n* = 6 ± sem) **C** and the percentage of cell death was determined by Annexin-V labeling and flow cytometry analysis (*n* = 5 ± sem) **D**. **E** CD34+ cells from JAK2^V617F^-positive MPN patient samples were plated for colony forming assay in semi-solid medium supplemented or not with ruxolitinib (250 nM) and chloroquine (1 µM) or SAR405 (3 µM). After 14 days, clonogenic potential was assessed by counting the number of BFU-E clones per dish. Data are represented as percentage of control (*n* = 14 ± sem). **F**, **G**. JAK2^WT^ HL-60 cells were treated or not with ruxolitinib (1 µM) in the presence or absence of chloroquine (20 µM) or SAR405 (3 µM). After 3 days, the number of cells was assessed by trypan blue exclusion counting (*n* = 3 ± sem) **F** and the percentage of cell death was determined by Annexin-V labeling and flow cytometry analysis (*n* = 3 ± sem) **G**. **H**, **I** CD34+ cells from JAK2^WT^ healthy donor samples treated or not with ruxolitinib (1 µM) in the presence or absence of chloroquine (10 µM) or SAR405 (3 µM). After 2 days, the number of cells was assessed by trypan blue exclusion counting (*n* = 5 ± sem) **H** and the percentage of cell death was determined by Annexin-V labeling and flow cytometry analysis (*n* = 5 ± sem) **I**. **J** CD34+ cells from JAK2^WT^ healthy donor samples were plated for colony forming assay in semi-solid medium supplemented as indicated. After 14 days, clonogenic potential was assessed by counting the number of BFU-E clones per dish. Data are represented as percent of control (*n* = 10 ± sem).

We next sought to determine whether ruxolitinib-induced autophagy is cytotoxic or cytoprotective in JAK2^V617F^-expressing cells. Therefore, we first assessed the impact of combining ruxolitinib with different autophagy inhibitors (e.g., chloroquine and SAR405) on cell number and viability in JAK2^V617F^ models. We first checked the effectiveness of autophagosome formation inhibition by SAR405 [20], a lipid kinase VPS34 inhibitor. As shown in Supplementary Fig. 2A, the levels of LC3B-II were strongly reduced in HEL cells treated with SAR405 in combination with bafilomcycin, compared to where bafilomcycin had been used on its own. We then observed that ruxolitinib combined with SAR405 or chloroquine significantly decreased cell number and increased cell death compared to ruxolitinib alone in HEL and SET-2 cell lines (Fig. 2A, B). Moreover, ruxolitinib combination with chloroquine or SAR405 compared to the drugs alone also led to a significant reduction in MPN patient cells number (Fig. 2C) but not of cell death (Fig. 2D). This data suggest that ruxolitinib combined with autophagy inhibition may inhibit growth of JAK2 mutant patient cells rather than directly killing them. In addition, to study the impact of ruxolitinib treatment in the presence or absence of autophagy inhibitors on the ability of MPN patients’ single cells to form erythroid clones, we performed a colony formation assay. We observed that while ruxolitinib had a limited efficacy at reducing clonogenic properties of MPN patient cells, its combination with either chloroquine or SAR405 strongly decreased the number of erythroid clones compared with the drugs administered alone (Fig. 2E and Supplementary 2B). Conversely, those results were not observed with JAK2 WT cells. Indeed, ruxolitinib combined with both autophagy inhibitors neither increased JAK2 WT HL-60 (Fig. 2F, G) or normal hematopoietic cell response to ruxolitinib (Fig. 2H, I) nor reduced the number of erythroid clones formed from normal hematopoietic cells, compared to ruxolitinib treatment alone (Fig. 2J).

Altogether, our results show that ruxolitinib induces a cytoprotective autophagy in JAK2^V617F^ cells and not in JAK2 WT cells and that its pharmacological inhibition only sensitizes JAK2^V617F^ cells to ruxolitinib. These findings suggest that autophagy inhibition could improve the limited efficacy of ruxolitinib observed in clinics.

### Protein-phosphatase 2 A (PP2A) is involved in ruxolitinib-induced autophagy

**Fig. 3:**
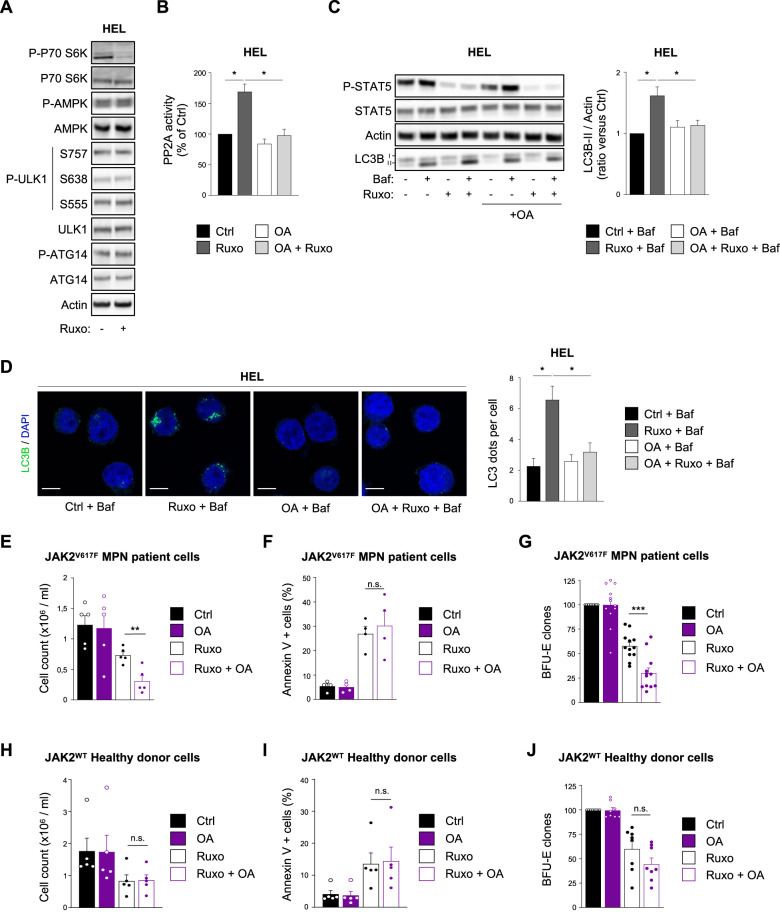
Ruxolitinib-induced autophagy relies on PP2A phosphatase activation. **A** HEL cells were treated with ruxolitinib (1 µM) for 2 h and processed for western blot analysis of the indicated proteins. Actin was used as a loading control. **B** HEL cells were treated with ruxolitinib (1 µM) in the presence or absence of the PP2A inhibitor okadaic acid (OA) (5 nM) for 2 h and processed for PP2A activity measurement by immuno-colorimetric assay. Data are represented as percentage of control (*n* *=* 3 ± s.e.m.). **C**, **D** HEL cells were treated with ruxolitinib (1 µM) and OA (5 nM) for 2 h in the presence or absence of bafilomycin (25 nM) to monitor autophagy flux. **C** Cells were processed for western blot analysis of P-STAT5, STAT5, and LC3B. Actin was used as a loading control. Histograms represent the LC3B-II/actin ratio obtained by densitometric analysis (*n* = 4 ± sem). **D** Cells were stained for LC3B and analyzed by confocal microscopy. Graphs represent the number of LC3B dots per cell (*n* = 3 ± sem). Scale bar: 10 µm. **E**, **F** CD34+ cells obtained from JAK2^V617F^-positive MPN patient samples were treated or not with ruxolitinib (1 µM) in the presence or absence of OA (5 nM). After 2 days, the number of cells was assessed by trypan blue exclusion counting (*n* = 5 ± sem) **E** and the percentage of cell death was determined by Annexin-V labeling and flow cytometry analysis (*n* = 4 ± sem) **F**. **G** CD34+ cells from JAK2^V617F^-positive MPN patient samples were plated for colony forming assay in semi-solid medium supplemented or not with ruxolitinib (250 nM) and OA (5 nM). After 14 days, the clonogenic potential was assessed by counting the number of BFU-E clones per dish. Data are represented as percentage of control (*n* *=* 12 ± sem). **H**, **I** CD34+ cells obtained from JAK2^WT^ healthy donor samples were treated or not with ruxolitinib (1 µM) in the presence or absence of OA (5 nM). After 2 days, the number of cells was assessed by trypan blue exclusion counting (*n* = 5 ± sem) **H** and the percentage of cell death was determined by Annexin-V labeling and flow cytometry analysis (*n* = 4 ± sem) **I**. **J** CD34+ cells from JAK2^WT^ healthy donor samples were plated for colony forming assay in semi-solid medium supplemented or not with ruxolitinib (250 nM) and OA (5 nM). After 14 days, clonogenic potential was assessed by counting the number of BFU-E clones per dish. Data are represented as percent of control (*n* = 8 ± sem).

Following this, we investigated the molecular mechanisms by which ruxolitinib induces autophagy in JAK2^V617F^ MPN cells. We first evaluated the potential role of mTORC1 and AMPK, two major signaling pathways involved in autophagy regulation. As previously described in the literature, mTORC1 was inhibited upon ruxolitinib treatment [17], as shown by a decrease in the phosphorylated form of P70S6K, one of its bona fide substrates (Fig. 3A). As mTORC1 is a well-known negative regulator of autophagy [21], its inhibition appeared therefore to be a potential candidate responsible for the autophagy induction observed upon ruxolitinib treatment. mTORC1 inhibits autophagy through phosphorylation of ULK1 kinase on serines 757 and 638 [22, 23]. However, as shown in Fig. 3, these ULK1 phosphosites were not modified upon ruxolitinib treatment (Fig. 3A). Moreover, the phosphorylation of AMPK and its direct downstream target, the ULK1 serine 555, were not modified through ruxolitinib treatment either (Fig. 3A). These results suggest that ruxolinitib-dependent autophagy induction is due neither to mTORC1 inhibition nor to AMPK activation.

Previous studies described that PP2A phosphatase may be repressed downstream JAK2^V617F^ in murine cell lines, as JAK2 inhibition by JAK2 inhibitor type I increases its activity [24]. Moreover, PP2A has also been shown to trigger autophagy upon amino acid starvation in human fibrosarcoma cell lines [25]. Based on these observations, we hypothesized that PP2A could be involved downstream JAK2^V617F^ in ruxolitinib-induced autophagy. To address this question, we used okadaic acid (OA), a well-known inhibitor of PP2A, at a low concentration (5 nM) to specifically target PP2A [26], and we first monitored PP2A activity using an immunocolorimetric assay [24]. As shown in Fig. 3B and in accordance with data from the literature, ruxolitinib treatment significantly increased PP2A activity in HEL cells. Moreover, OA prevented the ruxolitinib-induced PP2A activity thus indicating that OA, at this concentration, efficiently inhibits PP2A activity. We then used OA to modulate PP2A activity and evaluated whether this phosphatase is involved in ruxolitinib-induced autophagy. Interestingly, OA abrogated the increase of LC3-II accumulation and autophagosomes number (Fig. 3C, D and Supplementary Fig. 3A, B) triggered by ruxolitinib in both JAK2^V617F^ cell lines. Furthermore, FTY720, a PP2A-activating drug [24, 27], increased autophagy flux (Supplementary Figure 3C) and autophagosomes number (Supplementary Fig. 3D). Collectively, these data indicate that PP2A is activated upon JAK2 inhibition and is responsible, at least in part, for the autophagy induction observed upon ruxolitinib treatment, drawing a connection between ruxolitinib-dependent PP2A activation and autophagy induction for the first time.

### Protein-phosphatase 2 A (PP2A) inhibition enhances ruxolitinib cytotoxicity

Since targeting autophagy in JAK2^V617F^ model enhances the effects of ruxolitinib (Fig. 2), inhibiting PP2A activity, which is responsible for autophagy induction, could also represent an interesting strategy for improving the efficacy of ruxolitinib. To investigate this point, we treated primary cells from MPN patients with OA alone, to inhibit PP2A activity, or in combination with ruxolitinib. Similar to autophagy inhibition (Fig. 2C–E), this new drug combination significantly increased the impact of ruxolitinib on MPN cells number (Fig. 3E), did not modify cell death (Fig. 3F) and strongly reduced erythroid clone number (Fig. 3G) compared to ruxolitinib alone. Importantly, OA did not potentiate ruxolitinib effects on normal hematopoietic cells from healthy donors (Fig. 3H-J), as previously described for autophagy inhibition (Fig. 2H, I). Consistent with the data obtained with OA, more selective PP2A inhibitor LB-100 [28] also prevented ruxolitinib-induced autophagy (Supplementary Figures 3E,F) and increased the effect of ruxolitinib on the number of erythroid clones in MPN patients’samples (Supplementary Figure 3G). These results confirm that PP2A-dependent autophagy activation by ruxolitinib limits the effects of this TKI.

### Targeting autophagy enhances the survival of ruxolitinib-treated mice engrafted with JAK2^V617F^ cells

**Fig. 4:**
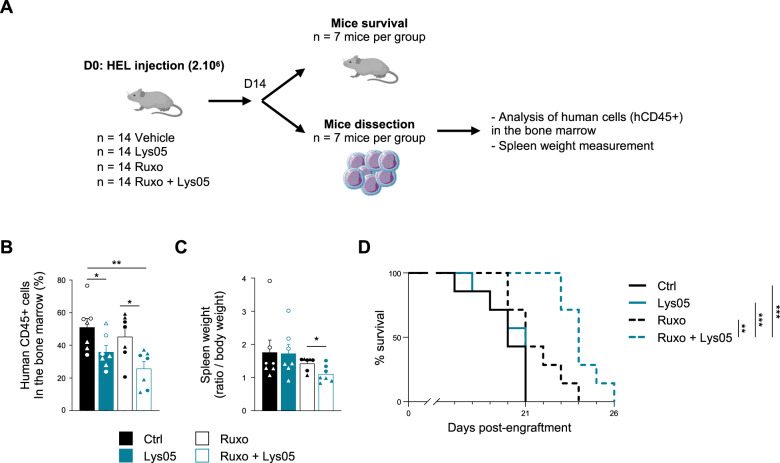
Targeting autophagy increases survival of ruxolitinib-treated mice xenografted with JAK2^V617F^ cells. **A** NSG mice (*n* = 14 mice per group) engrafted with 2.10^6^ HEL cells by intravenous injection were treated with vehicle or ruxolitinib (120 mg/kg/day) by oral gavage in combination or not with Lys05 (32 mg/kg/day) by intraperitoneal injection. **B** Fourteen days post-engraftment, seven mice per group were dissected to analyze the percentage of viable human cells (Annexin-V-, hCD45 + ) in the bone marrow by flow cytometry (*n* = 7 ± sem). **C** Their spleens were collected and weighted. Graphs represent spleen weight related to body weight for each mouse individually (left panel; *n* = 7 ± sem). **D** The remaining seven mice per group were used for overall survival analysis and Kaplan-Meier survival curves of engrafted mice are shown (*n* = 7 ± sem).

To test the functional relevance of targeting autophagy in combination with ruxolitinib, we subjected NOD-SCID-gamma (NSG) immunodeficient mice engrafted with HEL cells to a daily treatment of Lys05 (Fig. 4A), a more potent autophagy inhibitor than chloroquine [29, 30]. We first validated that this lysosomotropic agent phenocopied the results obtained in vitro with other autophagy inhibitors regarding cell number, viability and clonogenic properties. As shown in Supplementary Figure 4A, Lys05 reduced HEL cell number independently of ruxolitinib inhibition. This effect, even if there was a tendency, was not significant on the number of erythroid clones from primary MPN patient cells (Supplementary Figure 4B). This slight difference of Lys05 sensitivity is probably due to the fact that cell lines are highly proliferative compared to primary cells that grow slowly when cultivated in vitro. However, as observed with chloroquine and SAR405, Lys05 treatment strongly enhanced the cytotoxic effects of ruxolitinib in JAK2^V617F^ cell lines (Supplementary Figure 4A) and robustly reduced the number of erythroid clones from MPN patients’ cells (Supplementary Figure 4B). As shown for chloroquine, Lys05 modified neither the response of JAK2 WT HL-60 cells to ruxolitinib (Supplementary Figure 4C) nor the number of clones from normal hematopoietic cells (Supplementary Figure 4D).

In addition, in vivo experiments demonstrated that Lys05 improved the response to ruxolitinib. Indeed, mice engrafted with HEL cells and treated with Lys05 plus ruxolitinib displayed a higher reduction of the percentage of human CD45+ cells present in the bone marrow (Fig. 4B). Accordingly, as splenomegaly reduction is a hallmark of therapeutic efficacy, we measured the spleen weight of mice treated with the drug combination and we observed a decrease in weight compared to ruxolitinib treatment alone (Fig. 4C and Supplementary Fig. 4E). Moreover, treatment with a combination of ruxolitinib and Lys05 was significantly more efficient in enhancing the survival rate of mice than JAK2 inhibitor alone (Fig. 4D).

In summary, our data reveal for the first time that autophagy inhibition potentiates the effect of ruxolitinib and improves the overall survival in vivo.

Altogether, by providing a better understanding of the molecular and cellular mechanisms triggered by ruxolitinib, our findings have identified the PP2A-autophagy axis as a novel therapeutic target in MPN. This indicates that combining ruxolitinib with either autophagy or PP2A inhibitors could improve ruxolitinib response in MPN patients.

## Discussion

In this work, we show that JAK2 inhibitors such as ruxolitinib induced autophagy in JAK2^V617F^ cells. Ruxolitinib has been previously reported as an autophagy inducer in one JAK2^V617F^ cell line [18]. Our results further demonstrate that autophagy is induced upon ruxolitinib treatment in two cell lines expressing JAK2^V617F^ but also in primary JAK2^V617F^ patient cells. We also observed that the ruxolitinib-induced autophagy is cytoprotective. Furthermore, the autophagy induction was not found in their WT counterpart, indicating that targeting autophagy in MPN JAK2^V617F^ patients may selectively target malignant hematopoietic cells. In vivo experiments confirmed those data as autophagy inhibition enhanced ruxolitinib efficacy and overall mice survival. Otherwise, ruxolitinib is not the unique TKI able to induce autophagy. Indeed, imatinib and crizotinib have the same effect in BCR-ABL1 chronic myeloid leukemia and ALK-anaplastic cell lymphoma, respectively [16, 31]. Autophagy has been shown to play an important role in therapeutic resistance in hematological malignancies (for review see [32–34]). Studies have reported that imatinib induces autophagy in leukemic stem cells (LSCs) from CML and that autophagy inhibition in combination with TKI leads to LSC elimination [29, 35]. These pre-clinical studies, especially the pioneer one made by Bellodi et al. in 2009 [16], provided the rationale to evaluate into the clinic whether inhibiting autophagy with hydroxychloroquine (HCQ) a derivative of chloroquine, in association with conventional therapies (either chemotherapies or targeted therapies) improve patients’ therapeutic response. However, several clinical trials reported that HCQ does not consistently inhibit autophagy in patients when used at tolerated doses [36–38] explaining why HCQ showed limited efficacy. Therefore, this combined with the fact that the effect of HCQ on cancer cells can be autophagy-independent supported the development of more specific and more potent autophagy inhibitors. New ones targeting, for example, VPS34 [20] or ULK1 [39] have been developed and pre-clinical studies have shown promising results with the ULK1 inhibitor, the MRT403, currently tested in a phase 1/2 clinical trial alone or in combination with MAPK kinase inhibitors in patients with RAS/MAPK pathway mutation (ClinicalTrials.gov; NCT04892017). Overall, these data indicate that autophagy represents a promising therapeutic target in several cancers especially in hematological malignancies expressing specific oncogenes.

However, the molecular mechanisms underlying the autophagy-dependent resistance are still understudied. JAK2-mutated MPN cells display an elevated glycolysis and an increased mitochondrial metabolism [40] necessary to support their high demand in energy. Interfering with their exacerbated metabolism reduced cell proliferation and survival, effects that were amplified when combined with ruxolitinib. However, ruxolitinib probably affects mitochondrial respiration modestly, explaining its moderate impact on cell proliferation and death [40]. These results suggest that alternative mechanisms that support metabolic pathways occur upon treatment with ruxolitinib. In this study, we demonstrated that ruxolitinib induces autophagy, a well-known regulator of cell metabolism [41] and especially of oxidative metabolism in hematological malignancies [42]. We can speculate that this autophagy could therefore limit drug efficacy in vitro and in vivo through its ability to sustain cell metabolism. Future research is warranted to discern whether and how autophagy maintains elevated oxidative phosphorylation upon ruxolitinib treatment and to explain how autophagy participates in ruxolitinib resistance.

Here, we identified PP2A as an important modulator downstream JAK2^V617F^. Upon activation, wild-type JAK2 was described to directly interact with and deactivate PP2A [43], indicating that PP2A exerts a negative role on JAK2-mediated signal transduction. Mutated JAK2 was also shown to deactivate PP2A in murine Ba/F3-JAK2^V617^ cells through the PI3Kγ-PKC-induced phosphorylation of the PP2A inhibitor SET [24]. In this study, PP2A activation with PP2A activating drug (PAD) decreases leukemic burden and significantly extends overall mice survival in an immunodeficient mouse model for leukemic cell engraftment, indicating that PADs have a strong anti-leukemic activity. These findings stand in contrast to what we describe. Indeed, we demonstrated that inhibiting PP2A in MPN patients’ cells in combination with ruxolitinib is more efficient in reducing their clonogenic properties than ruxolitinib alone, indicating that the PP2A activation induced by JAK2 inhibition favors the proliferation and survival of MPN cells. PP2A is a family of more than two hundred heterotrimeric complexes composed of a combination of one catalytic subunit, one scaffold subunit and one of many possible B regulatory subunits. Different B subunits were shown to display the opposite impact on mutated JAK2. Indeed, the B56α subunit of the PP2A complex repressed JAK2^V617F^ activity, whereas the B56γ one favors JAK2^V617F^ phosphorylation [44]. Therefore, different PP2A complexes have opposite role in MPN and we could speculate that is it not the same PP2A complex that is engaged upon ruxolitinib treatment as the PP2A complex that is activated by PADs. We can also consider that it is not the same PP2A complexes that are inactivated by wild-type or mutated JAK2, which could explain why autophagy is only induced upon treatment with ruxolitinib in mutated cells.

In addition, different PP2A complexes are implicated in autophagy regulation. On one hand, PP2A-PP2R3B was shown to dephosphorylate ATG4B, thus enabling LC3 processing and therefore autophagosome expansion [45]. Then again, by dephosphorylating ULK1 the PP2A-B55α complex relieves an inhibitory phosphorylation site required for both its activation and autophagosome formation [25]. For instance, the activity of different PP2A complexes, independent of mTORC activity, positively modulates autophagy by acting either at early or late steps of the autophagy process. PP2A can also control autophagy at the transcriptional level. In fact, the dephosphorylation of the transcription factor TFEB by PP2A induces its nuclear localization followed by the transcription of genes involved in autophagy and lysosomal functions [46]. Thus, an interesting question could be whether ruxolitinib selectively regulates the activity of PP2A complexes containing specific B-regulatory subunits that mediate its effect on autophagy.

Further studies are therefore needed to identify which specific subsets of PP2A are responsible for ruxolitinib-induced autophagy and which proteins are involved in regulating autophagy. Specifically targeting the correct PP2A complex rather than all PP2A complexes is necessary to improve therapy efficacy.

Interestingly, similar to autophagy inhibition, metformin increases ruxolitinib efficacy in cell lines harboring the JAK2^V617F^ mutation [44]. As metformin has recently been shown to inhibit autophagy in AML cell lines [42], we can therefore speculate that metformin enhances ruxolitinib’s negative impact on cell growth through its inhibitory action upon autophagy. Hence, metformin could, as an autophagy inhibitor, represent a new therapeutic option to be combined with ruxolitinib to treat JAK2^V617F^ MPN patients.

Ruxolitinib response is often short-lived due to the development of resistance to it, and therefore combination therapy appeared to be essential in improving the therapeutic response of MPN patients expressing the mutated JAK2^V617F^. Our findings demonstrated that autophagy is induced upon JAK2 inhibition by TKI and that inhibiting this degradative pathway improves ruxolitinib efficacy both in vitro and in vivo. Furthermore, we found that PP2A activation is involved in ruxolitinib-induced autophagy, and our study is the first to establish a connection between ruxolitinib, PP2A activation and autophagy induction in the same cellular model. Thus, targeting this phosphatase together with ruxolitinib impedes cell proliferation and the survival of JAK2-mutated cells.

Collectively, these data offer a rationale for clinical trials to assess the efficacy of JAK2 and PP2A-dependent autophagy inhibition in MPN patients expressing JAK2^V617F^ mutant.

## Supplementary information

Supplementary file

## Data Availability

The datasets generated during and/or analyzed during the current study are available from the corresponding authors upon reasonable request.
