# Supplementary material for: Targeting PP2A-dependent autophagy enhances sensitivity to ruxolitinib in JAK2V617F myeloproliferative neoplasms
Source: Blood Cancer J. 2023 Jul 10;13(1):106. doi: 10.1038/s41408-023-00875-x (PMC10330179; doi:10.1038/s41408-023-00875-x)
Supplement: Supplementary file 1 — Supplementary file [file 41408_2023_875_MOESM1_ESM.pdf]

Supplementary Information

Targeting PP2A-dependent autophagy enhances sensitivity to ruxotlitinib in JAK2^{V617F} myeloproliferative neoplasms

Courdy et al.

Table of Contents:

- Supplementary Table 1

- Supplementary Figures 1-4

	ID study	From	$JAK2^{V617F}$ allelic burden at diagnosis	Follow-up treatment
$JAK2^{V617F}$-positive MPN patients	12	Toulouse University Hospital	"Intermediate"	Phlebotomy
	13		45%	Phlebotomy
	16		60%	Phlebotomy
	17		36%	Phlebotomy
	18		23%	Phlebotomy
	19		45%	Phlebotomy
	20		13%	Phlebotomy
	22		45%	Phlebotomy
	23		87%	Phlebotomy
	29		85%	Phlebotomy + pegylated interferon
	31		43%	Phlebotomy
	32		<10%	Phlebotomy
	33		27%	Phlebotomy
	34		10%	Phlebotomy
	35		39%	Phlebotomy
	36		>50%	Phlebotomy
	37		21%	Phlebotomy
	38		23%	Phlebotomy
	39		9%	Phlebotomy
	40		<10%	Phlebotomy
	41		Not referred	Phlebotomy
	42		7%	Phlebotomy
	43		34%	Phlebotomy + aspirin
	44		67%	Phlebotomy + aspirin
	46		20%	Phlebotomy + aspirin
$JAK2^{WT}$ healthy donors	A	EFS, Toulouse	None	None
	B		None	None
	C		None	None
	D		None	None
	E		None	None
	F		None	None
	G		None	None
	H		None	None
	I		None	None
	J		None	None

Supplementary Table 1. Primary MPN patient and healthy donor characteristics.

Figure S1

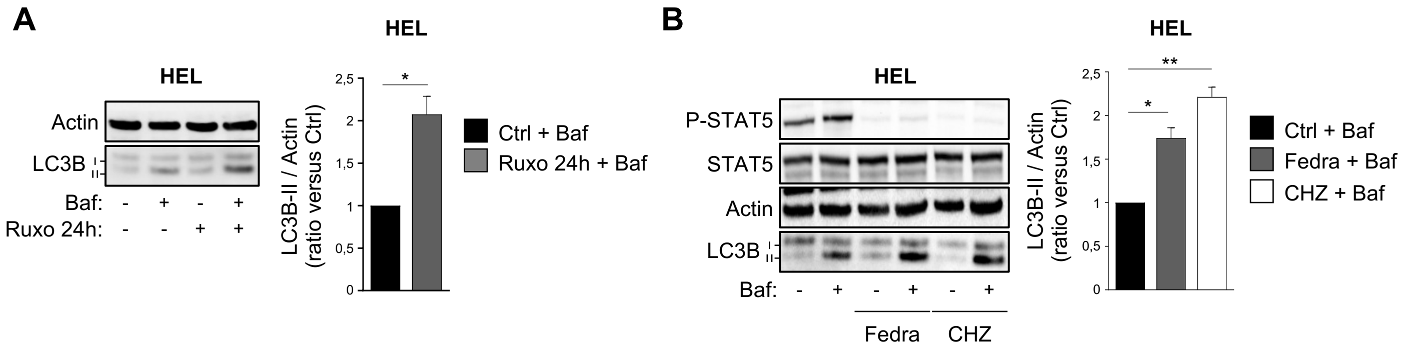
A
HEL
Actin
LC3B
I
II
Baf: - + - +
Ruxo 24h: - - + +
HEL
*
LC3B-II / Actin (ratio versus Ctrl)
2,5
2
1,5
1
0,5
0
Ctrl + Baf
Ruxo 24h + Baf
B
HEL
P-STAT5
STAT5
Actin
LC3B
I
II
Baf: - + - + - +
Fedra
CHZ
HEL
**
*
LC3B-II / Actin (ratio versus Ctrl)
2,5
2
1,5
1
0,5
0
Ctrl + Baf
Fedra + Baf
CHZ + Baf

Figure S2

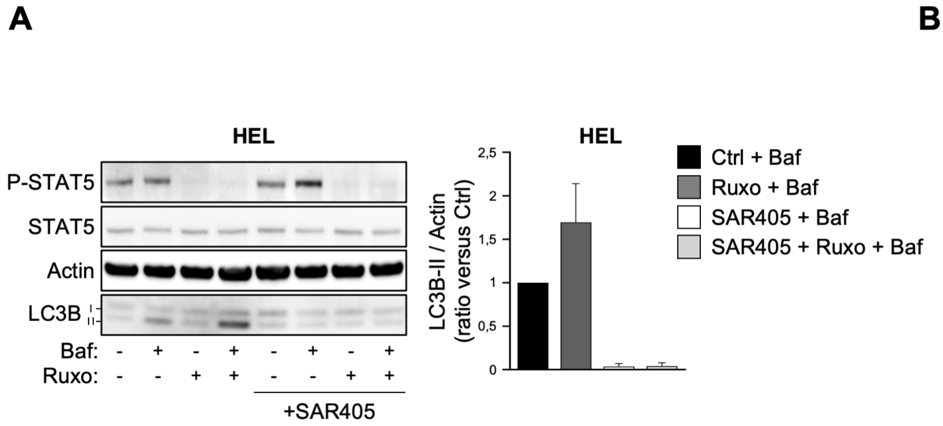
A
HEL
P-STAT5
STAT5
Actin
LC3B
I
II
Baf:
Ruxo:
+SAR405
HEL
LC3B-II / Actin
(ratio versus Ctrl)
2,5
2
1,5
1
0,5
0
Ctrl + Baf
Ruxo + Baf
SAR405 + Baf
SAR405 + Ruxo + Baf

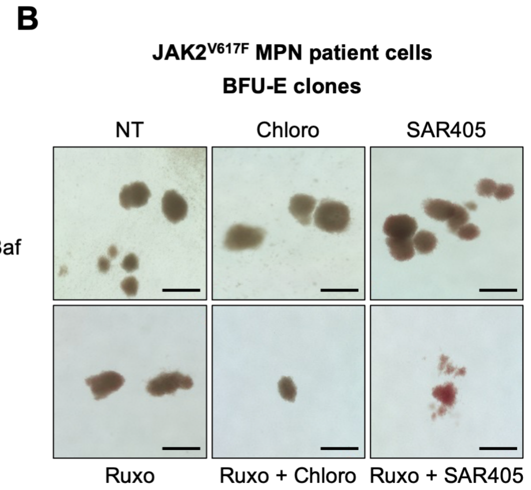
B
JAK2V617F MPN patient cells
BFU-E clones
NT
Chloro
SAR405
Ruxo
Ruxo + Chloro
Ruxo + SAR405

Figure S3

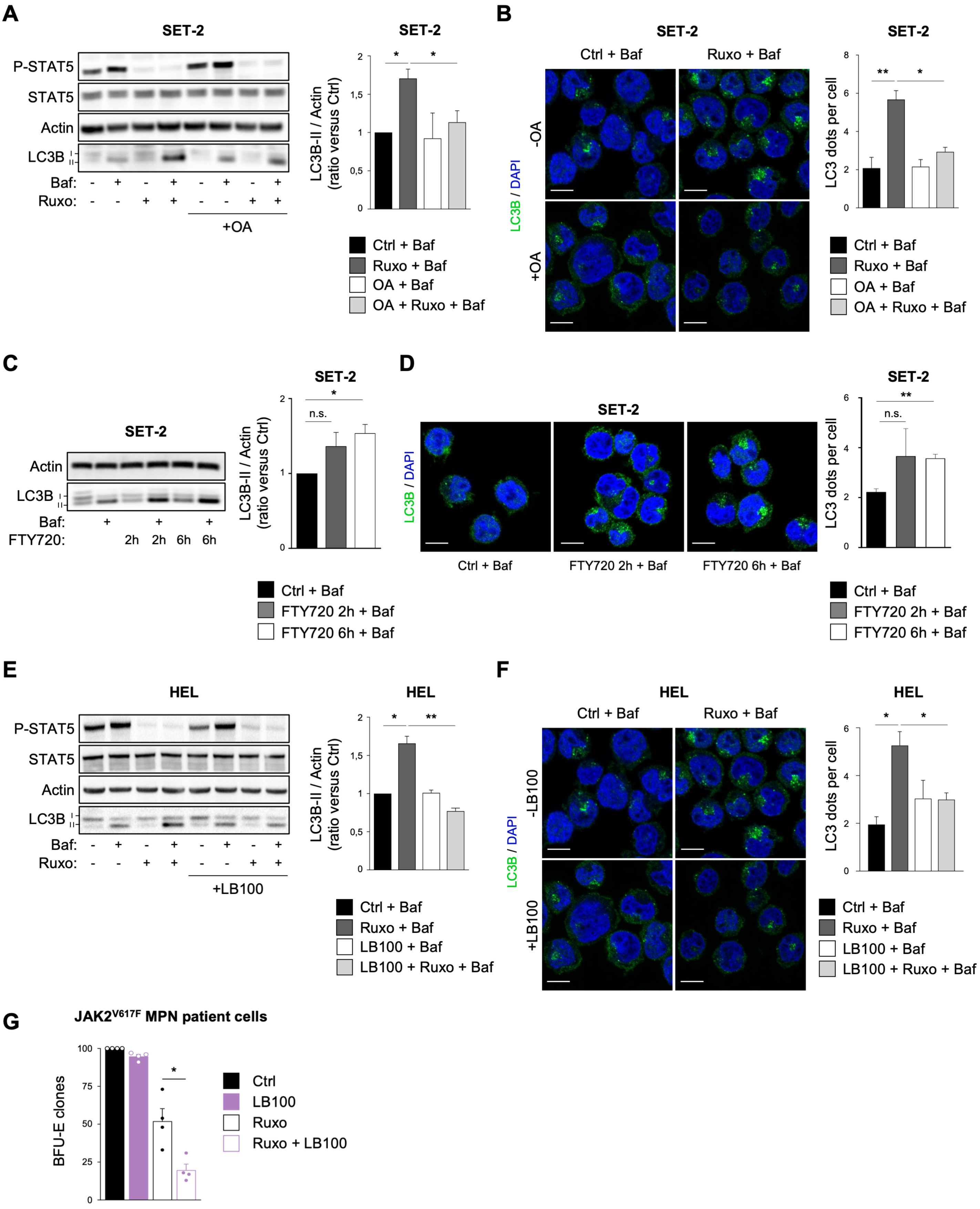
A
SET-2
P-STAT5
STAT5
Actin
LC3B
Baf:
Ruxo:
+OA
LC3B-II / Actin (ratio versus Ctrl)
Ctrl + Baf
Ruxo + Baf
OA + Baf
OA + Ruxo + Baf
B
SET-2
Ctrl + Baf
Ruxo + Baf
-OA
+OA
LC3B / DAPI
LC3 dots per cell
C
SET-2
Actin
LC3B
Baf:
FTY720:
2h
6h
n.s.
FTY720 2h + Baf
FTY720 6h + Baf
D
SET-2
FTY720 2h + Baf
FTY720 6h + Baf
E
HEL
+LB100
LB100 + Baf
LB100 + Ruxo + Baf
F
HEL
-LB100
+LB100
G
JAK2V617F MPN patient cells
BFU-E clones
Ctrl
LB100
Ruxo
Ruxo + LB100

Figure S4

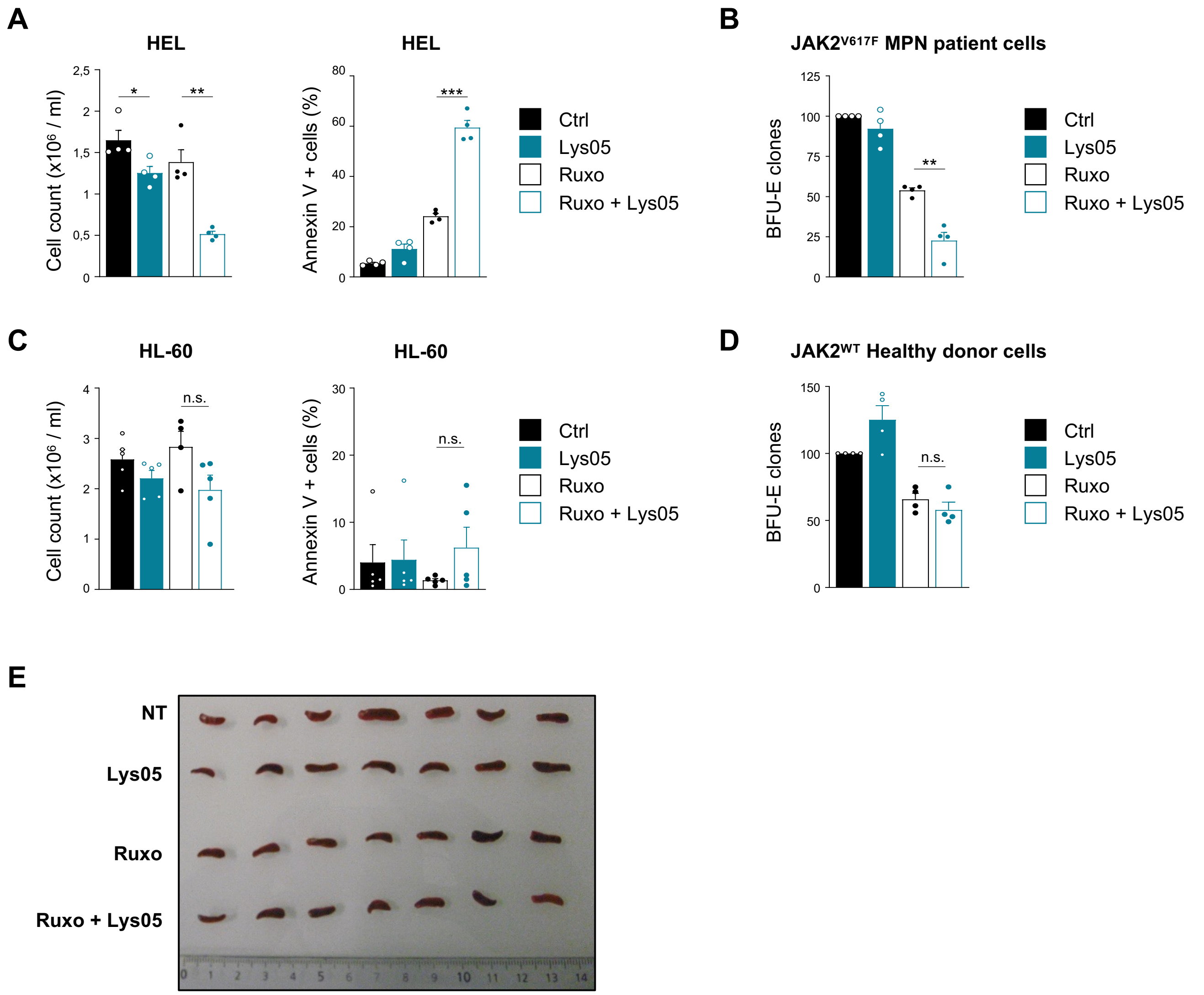
A
HEL
Cell count (x10⁶ / ml)
2,5
2
1,5
1
0,5
0
*
**
HEL
Annexin V + cells (%)
80
60
40
20
0
***
Ctrl
Lys05
Ruxo
Ruxo + Lys05
B
JAK2V617F MPN patient cells
BFU-E clones
125
100
75
50
25
0
**
Ctrl
Lys05
Ruxo
Ruxo + Lys05
C
HL-60
Cell count (x10⁶ / ml)
4
3
2
1
0
n.s.
HL-60
Annexin V + cells (%)
30
20
10
0
n.s.
Ctrl
Lys05
Ruxo
Ruxo + Lys05
D
JAK2WT Healthy donor cells
BFU-E clones
150
100
50
0
n.s.
Ctrl
Lys05
Ruxo
Ruxo + Lys05
E
NT
Lys05
Ruxo
Ruxo + Lys05

Figure S1. Ruxolitinib increases autophagy in JAK2^{V617F} cells. **A.** HEL cells were treated with ruxolitinib (1µM) for 24 hours in the presence or absence of bafilomycin (25nM) over 2 hours to monitor autophagy flux and processed for western blot analysis of LC3B. Actin was used as a loading control. Graphs represent the LC3B-II/actin ratios obtained by densitometric analysis (n=3 ± s.e.m.). **B.** HEL cells were treated with fedratinib (1µM) or CHZ868 (500nM) for 2 hours in the presence or absence of bafilomycin (25nM) to monitor autophagy flux and processed for western blot analysis of P-STAT5, STAT5 and LC3B. Actin was used as a loading control. Graphs represent the LC3B-II/actin ratios obtained by densitometric analysis (n=3 ± s.e.m.).

Figure S2. Autophagy inhibition enhances ruxolitinib efficacy in JAK2^{V617F} cells. **A.** HEL cells were treated with ruxolitinib (1µM) and SAR405 (3µM) for 2 hours in the presence or absence of bafilomycin (25nM) to monitor autophagy flux and processed for western blot analysis of P-STAT5, STAT5 and LC3B. Actin was used as a loading control. Graphs represent the LC3B-II/actin ratios obtained by densitometric analysis (n=2 ± s.e.m.). **B.** Representative picture of BFU-E clones after 14 days of patients' CD34+ MPN cells cultured in semi-solid medium supplemented or not with ruxolitinib (250nM) and chloroquine (1µM) or SAR405 (3µM). Scale bar: 300µm.

Figure S3. PP2A inhibition enhances ruxolitinib efficacy in JAK2^{V617F} cells. **A-B.** SET-2 cells were treated with ruxolitinib (1µM) and OA (5nM) for 2 hours in the presence or absence of bafilomycin (25nM) to monitor autophagy flux. (**A**) Cells were processed for western blot analysis of P-STAT5, STAT5 and LC3B. Actin was used as a loading control. Graphs represent the LC3B-II/actin ratios obtained by densitometric analysis (n=4 ± s.e.m.). (**B**) Cells were stained for LC3B and analyzed by confocal microscopy. Graphs represent the number of LC3B dots per cell (n=4 ± s.e.m.). Scale bar: 10µm. **C-D.** SET-2 cells were treated with FTY720 (2.5µM) for 2 or 6 hours in the presence or absence of bafilomycin (25nM) to monitor autophagy flux. (**C**) Cells were processed for western blot analysis of LC3B. Actin was used

as a loading control. Graphs represent the LC3B-II/actin ratios obtained by densitometric analysis (n=3 ± s.e.m.). (**D**) Cells were stained for LC3B and analyzed by confocal microscopy. Graphs represent the number of LC3B dots per cell (n=3 ± s.e.m.). Scale bar: 10µm. **E-F.** HEL cells were treated with ruxolitinib (1µM) and LB100 (2.5µM) for 2 hours in the presence or absence of bafilomycin (25nM) to monitor autophagy flux. (**E**) Cells were processed for western blot analysis of P-STAT5, STAT5 and LC3B. Actin was used as a loading control. Graphs represent the LC3B-II/actin ratios obtained by densitometric analysis (n=3 ± s.e.m.). (**F)** Cells were stained for LC3B and analyzed by confocal microscopy. Graphs represent the number of LC3B dots per cell (n=3 ± s.e.m.). **F-G.** CD34+ cells from JAK2^{V617F}-positive MPN patient samples were plated for colony forming assay in semi-solid medium supplemented or not with ruxolitinib (250nM) and LB100 (1µM). (**F**) After 14 days, clonogenic potential was assessed by counting the number of BFU-E clones per dish. (**G**) Data are represented as percent of control (n=4 ± s.e.m.).

Figure S4. Autophagy inhibitor Lys05 enhances ruxolitinib efficacy in JAK2^{V617F} cells *in vitro* and *in vivo*. **A.** HEL cells were treated or not with ruxolitinib (1µM) in the presence or absence of Lys05 (5µM). After 3 days, the number of cells was assessed by trypan blue exclusion counting (left panel; n=4 ± s.e.m.) and the percentage of cell death was determined by Annexin-V labelling and flow cytometry analysis (right panel; n=4 ± s.e.m.). **B.** CD34+ cells from JAK2^{V617F}-positive MPN patient samples were plated for colony forming assay in semi-solid medium supplemented or not with ruxolitinib (250nM) and Lys05 (1µM). After 14 days, clonogenic potential was assessed by counting the number of BFU-E clones per dish. Data are represented as percent of control (n=4 ± s.e.m.). **C.** JAK2^{WT} HL-60 cells were treated or not with ruxolitinib (1µM) in the presence or absence of Lys05 (5µM). After 3 days, the number of cells was assessed by trypan blue exclusion counting (left panel; n=5 ± s.e.m.) and the percentage of cell death was determined by Annexin-V labelling and flow cytometry analysis (right panel; n=5 ± s.e.m.). **D.** CD34+ cells from JAK2^{WT} healthy donor samples were plated for colony forming assay in semi-solid medium supplemented as indicated. After 14 days,

clonogenic potential was assessed by counting the number of BFU-E clones per dish. Data are represented as percent of control (n=4 ± s.e.m.). **E.** HEL cells were injected in NSG mice and after 14 days of the indicated treatments their spleens were collected.
